# Assessing uncertainty in sighting records: an example of the Barbary lion

**DOI:** 10.7717/peerj.1224

**Published:** 2015-09-01

**Authors:** Tamsin E. Lee, Simon A. Black, Amina Fellous, Nobuyuki Yamaguchi, Francesco M. Angelici, Hadi Al Hikmani, J. Michael Reed, Chris S. Elphick, David L. Roberts

**Affiliations:** 1 Mathematical Institute, University of Oxford, UK; 2 Durrell Institute of Conservation and Ecology, School of Anthropology and Conservation, University of Kent, Canterbury, Kent, UK; 3 Agence Nationale pour la Conservation de la Nature, Algiers, Algeria; 4 Department of Biological and Environmental Sciences, University of Qatar, Doha, Qatar; 5 Italian Foundation of Vertebrate Zoology (FIZV), Rome, Italy; 6 Office for Conservation of the Environment, Diwan of Royal Court, Sultanate of Oman; 7 Department of Biology, Tufts University, Medford, MA, USA; 8 Department of Ecology and Evolutionary Biology, Center for Conservation and Biodiversity, University of Connecticut, Storrs, CT, USA

**Keywords:** Data quality, Critically endangered, IUCN red list, Sighting record, Possibly extinct, Sighting uncertainty, Panthera leo, Extinct

## Abstract

As species become rare and approach extinction, purported sightings can be controversial, especially when scarce management resources are at stake. We consider the probability that each individual sighting of a series is valid. Obtaining these probabilities requires a strict framework to ensure that they are as accurately representative as possible. We used a process, which has proven to provide accurate estimates from a group of experts, to obtain probabilities for the validation of 32 sightings of the Barbary lion. We consider the scenario where experts are simply asked whether a sighting was valid, as well as asking them to score the sighting based on distinguishablity, observer competence, and verifiability. We find that asking experts to provide scores for these three aspects resulted in each sighting being considered more individually, meaning that this new questioning method provides very different estimated probabilities that a sighting is valid, which greatly affects the outcome from an extinction model. We consider linear opinion pooling and logarithm opinion pooling to combine the three scores, and also to combine opinions on each sighting. We find the two methods produce similar outcomes, allowing the user to focus on chosen features of each method, such as satisfying the marginalisation property or being externally Bayesian.

## Introduction

Rare species are often observed sporadically, meaning each sighting is rare and can greatly affect how conservation measures are applied (Roberts, Elphick & Reed, 2010). Time since last sighting is an important component when assessing the persistence of a species (Solow, 2005; Butchart, Stattersfield & Brooks, 2006); however, the exact timing of the last sighting itself may be uncertain due to the quality of sightings towards the end of a record (Jarić & Roberts, 2014). Incorrect declaration of extinction is not uncommon. Scheffers et al. (2011) identified 351 rediscovered species over the past 122 years (104 amphibians, 144 birds, and 103 mammals). Alternatively, a species could persist indefinitely in a state of purgatory as Critically Endangered (Possibly Extinct), thus incurring the costs associated with this status (McKelvey, Aubry & Schwartz, 2008)—for example the Ivory-billed Woodpecker (*Campephilus principalis*), see Jackson (2006), Sibley et al. (2006), Collinson (2007), Dalton (2010) and Roberts, Elphick & Reed (2010).

A growing number of models have been developed to infer extinction based on a sighting record (see Solow, 2005; Boakes, Rout & Collen, 2015 for reviews). However, it is not uncommon to find examples (Cabrera, 1932; Mittermeier, De Macedo Ruiz & Luscombe, 1975; Wetzel et al., 1975; Snyder, 2004) where the perceived acceptability, authenticity, validity or veracity of a sighting is attributed to an assessment of the observer (e.g., local hunters, ornithologists, collectors, field guides) based upon an arbitrary judgement of a third party and/or a perception of the conditions under which the sighting was made, rather than on a systematic consideration of the sighting. Further, there is a risk that only Western scientists are perceived competent to find and save threatened species (Ladle et al., 2009) which implies that the input of informed others (usually locals) is not valued.

Recently, several studies have developed methods of incorporating sighting uncertainty within the analysis of a sighting record (Solow et al., 2012; Thompson et al., 2013; Jarić & Roberts, 2014; Lee et al., 2014; Lee, 2014), with the most recent methods assigning probabilities of reliability to individual sightings (Jarić & Roberts, 2014; Lee et al., 2014). The outcomes from these models vary significantly as the sighting reliability varies. To ensure the correct application of these models, there is a need for an objective framework to evaluate ambiguous sightings (McKelvey, Aubry & Schwartz, 2008; Roberts, Elphick & Reed, 2010).

We present a formal structure to elicit expert opinions on sighting validity. To demonstrate this questioning technique we use the sighting record of the extinct North African Barbary lion (*Panthera leo leo*), for which a considerable amount of sighting data have recently been amassed from Algeria to Morocco (Black et al., 2013). The quality of these sightings varies from museum skins, to oral accounts elicited many years after the original sighting, some of which have proved controversial. Understanding the nature of lion sightings in North Africa will enable sophisticated extinction models to be applied to maximum effect. This will help inform the conservation of other extant very rare population, e.g., the Critically Endangered West African lion population (Angelici, Mahama & Rossi, 2015).

This paper quantifies the reliability probabilities using methods of eliciting expert opinion. We considered two approaches to ask experts about sighting reliability. First we asked for a probability that the sighting is true. This straightforward approach is the current technique (but sometimes only one expert is asked). Second, we asked the experts about three distinct factors which relate to sighting reliability (distinguishability, observer competence, verfiability). The result from combining these three aspects is compared to the result from asking the direct question. The three factors are combined using linear pooling, and logarithmic pooling (O’Hagan et al., 2009). The two different outcomes are then compared.

The questioning process is based on the work of Burgman et al. (2011) and McBride et al. (2012), where experts essentially provide a ‘best estimate’ and their upper and lower bounds on this best estimate. The expert opinions are combined by simply taking the mean of the best estimate, and bounding it by the means of the lower and upper bounds. We use this method, and again, we use linear pooling and logarithmic pooling methods. The advantages and disadvantages of each pooling technique are discussed.

The Barbary or Atlas lion of North Africa, ranged from the Atlas Mountains to the Mediterranean (the Mahgreb) during the 18th century. However, extensive persecution in the 19th century reduced populations to remnants in Morocco in the west, and Algeria and Tunisia in the east. The last evidence for the persistence of the Barbary lion in the wild is widely considered to be the animal shot in 1942 on the Tizi-n-Tichka pass in Morocco’s High Atlas Mountains (Black et al., 2013). However, later sightings have recently come to light from the eastern Mahgreb that push the time of last sighting to 1956. Previous analysis of these sighting records (where all sightings are considered valid) suggest that Barbary lions actually persisted in Algeria until 1958, ten years after the estimated extinction date of the western (Morocco) population (Black et al., 2013).

## Eliciting and Pooling Expert Opinions

### The questioning process

Experts can provide useful information which may be used as a variable in a model, as with extinction models. However, expert opinions often vary greatly. Previous work (Burgman et al., 2011; McBride et al., 2012) provide a method that elicits accurate results from experts, where the focus is on behavioural aspects so that peer pressure is minimised whilst still allowing group discussion. The method first requires experts to provide their independent opinion, which are then collated and anonymised. Second, the experts are brought together and provided the collated estimates, along with the original information provided. After experts have discussed the first round of estimates, they each privately provide revised estimates. We used this approach when asking five experts to provide responses to four questions Barbary lion sightings. While it is undisputed that several experts are better than one, there is a diminishing returns effect associated with large amounts of experts, with three to five being a recommended amount (Makridakis & Winkler, 1983; Clemen & Winkler, 1985).

All available information was provided for the last 32 alleged sightings of the Barbary lion. The sightings vary considerably, for example, one sighting is a photograph taken while flying over the Atlas mountains, another is lion observed by locals on a bus, and several other are shootings (see Supplemental Information 1). Using this information we followed the process provided by Burgman et al. (2011) and McBride et al. (2012). That is, the experts responded to each question with a value between 0 and 1 (corresponding to low and high scores) for each sighting. We refer to this value as the ‘best’ estimate. Additionally, for each question, experts provided an ‘upper’ and ‘lower’ estimate, and a confidence percentage (how sure the expert was that the ‘correct answer’ lay within their upper and lower bounds).

When an expert did not state 100% confidence that their estimate is within their upper and lower bounds, the upper and lower bounds were extended so that all bounds represented 100% confidence that the ‘correct answer’ lay within. This is a normalisation process to allow straightforward comparison across experts. For example, an expert may state that s/he is 80% confident that the ‘correct answer’ is between 0.5 and 0.9. We extend the bounds to represent 100% confidence, that is, 0.4 and 1.

Finally all experts were asked to anonymously assign a level of expertise to each of the other experts from 1 being low to 5 being high. These scores were used as a weighting so that reliability scores from those with greater perceived expertise had more influence in the model.

### The questions

Determining the probability that a sighting is true is very challenging—there are many factors and nuances which generally require experts to interpret how they influence the reliability of a sighting. First, experts were asked the straightforward question

(Q1) What is the probability that this sighting is of the taxon in question?

Typically, this is the extent of expert questioning. Second, to encourage experts to explicitly consider the issues surrounding identification, we asked three additional questions:

(Q2) How distinguishable is this species from others that occur within the area the sighting was made? Note that this is not based on the type of evidence you are presented with, i.e., a photo or a verbal account.
(Q3) How competent is the person who made the sighting at identifying the species, based on the evidence of the kind presented?
(Q4) To what extent is the sighting evidence verifiable by a third party?

These questions, and directions given to the experts as to how to respond, are provided in Supplemental Information 2. Responses to Q2, Q3 and Q4 provide a score for distinguishablity *D*, observer competency *O* and verifiability *V* respectively. We combine Q2 to Q4 in two different ways: linear pooling and logarithmic pooling. We now describe in detail what should be considered when allocating the scores.

Distinguishability score, *D*: that the individual sighting is identifiable from other taxa. This requires the assessor to consider other species within the area a sighting is made, and to question how likely is it that the taxon in question would be confused with other co-occurring taxa. In addition to the number of species with which the sighting could be confused, one should also take into consideration their relative population abundances in this estimate. For example, suppose there is video evidence which possibly shows a particular endangered species. But the quality of the video is such that it is uncertain whether the video has captured the endangered species, or a similar looking species which is more common. Based on known densities, home range size, etc. one might give this video a score of 0.2—that is, for every individual of the endangered species, there would be four of the more common species, or the more common species is four times more likely to be encountered.

Observer competency score, *O*: that the observer is proficient in making the correct identification. This requires the assessor to estimate, or presume, the ability of the observer to distinguish the taxon from other species. The assessment may be on the presumed ability of the observer to correctly identify the species they observe (e.g., limited for a three second view of a bird in flight, extent of the observers experience with the taxa, etc.), or based on the assessor’s own ability to identify the species from a museum specimen. Care should be taken to avoid unjustly favouring one observer over another.

Verifiability score, *V*: that the sighting evidence could be verified by a third party. This requires the assessor to determine the quality of the sighting evidence. For example a museum specimen or a photograph would score highly whereas a reported sighting where there is no evidence other than the person’s account would have a low score. Nonetheless, a recent observation has the opportunity for the assessor to return to the site and verify the sighting.

### Mathematical aggregation

To investigate whether the combined responses to Q2–Q4 provides a different outcome to asking simply Q1, we require an aggregation method. We use linear pooling and logarithm pooling (both described below and in O’Hagan et al. (2009)). Additionally, we require an aggregation method to pool the opinions from experts. One genre of aggregation is behavioural aggregation, which requires the experts to interact and converge upon one opinion. Alternatively, the experts provide separate opinions which are mathematically aggregated. Part of the questioning procedure involves a form of behavioural aggregation because experts discuss the sightings as a group. However, their final response is individual. As such, we also require mathematical aggregation.

The experts scores for each sighting need to be combined. For Q1 the pooled response that we used is the average of the ‘best’ estimates bounded by the averages of the extended lower and upper bounds (Burgman et al., 2011; McBride et al., 2012). For pooling expert opinions on Q2–Q4 (which are now represented as a single distribution for each expert, for each sighting), we use the same pooling technique that was used to combine the responses to Q2–Q4. That is, when Q2–Q4 are pooled linearly, the expert opinions are also pooled linearly, and similarly when Q2–Q4 are pooled logarithmically, the expert opinions are also pooled logarithmically. We now describe linear and logarithm pooling.

Consider the response to Q2, from one expert, for one sighting. For this single case, the expert has provided a best estimate, and two bounds (which are extended to encompass 100% of their confidence, see ‘The questioning process’). This opinion can be modelled as a triangle distribution *p*_1_(*θ*), with the peak at the best estimate, and the edges at the extended bounds. We pool this, together with the *p*_2_(*θ*) and *p*_3_(*θ*) from Q3 to Q4,

\begin{eqnarray*} p(\theta )=\sum _{i=1}^{n}{w}_{i}{p}_{i}(\theta ), \end{eqnarray*}

where *w_i_* is a weighting function such that $\sum _{i}^{n}{w}_{i}=1$ and, in this example, *n* = 3. Linear pooling is a popular method since it is simple, and it is the only combination scheme that satisfies the marginalisation property^1^1Suppose *θ* is a vector of uncertain quantities, and we are interested in just one element of the vector, *θ_i_*. According to marginalisation property, the combined probability is the same whether one combines the experts’ marginal distributions of *θ_i_*, or combines the experts’ joint distributions of the vector *θ* and then calculates the marginal distribution of *θ_i_* (Clemen & Winkler, 1999). (O’Hagan et al., 2009).

Alternatively, the consensus distribution *p*(*θ*) is obtained using a logarithmic opinion pool,

\begin{eqnarray*} p(\theta )=k\prod _{i=1}^{n}({p}_{i}(\theta ))^{{w}_{i}}, \end{eqnarray*}

where *w_i_* is the same weighting function as before, and *k* is a normalising constant that ensures ∫*p*(*θ*) = 1. The logarithmic opinion pool, unlike the linear opinion pool, is externally Bayesian^2^2Suppose we calculated *p*(*θ*) using a logarithmic pooling, but then learned some new information relevant to *θ*. Two choices are available. One is to use the information first to update the experts’ probability distribution *p_i_*(*θ*) and then combine them. The other is to use the information to update the combined *p*(*θ*) directly. A formula is externally Bayesian if the result is the same in both cases. and is also consistent with regard to judgements of independence (O’Hagan et al., 2009). However, it does not satisfy the marginalisation property which linear pooling does.

When pooling Q2–Q4, the *p_i_*, *i* = 1, 2, 3, are the responses for Q2–Q4, from each expert, for each sighting. We choose to weight each question equally, meaning *w_i_* = 1/3. When pooling the experts together, *p_i_*, *i* = 1, 2, …, 5, are the responses from each expert for the pooled responses Q2–Q4. We consider the case where each expert is weighted equally, meaning *w_i_* = 1/5, and the case where the experts are weighted by their scoring of each other; in our example *w*_1_ = 0.17, *w*_2_ = 0.13, *w*_3_ = 0.21, *w*_4_ = 0.21 and *w*_5_ = 0.28.

Linear pooling and logarithmic pooling are the simplest and most popular methods (Clemen & Winkler, 2007). Some complex models, such as a Bayesian combination, can be somewhat sensitive, leading to poor performance in some instances (Clemen & Winkler, 1999). In fact many studies (Seaver, 1978; Ferrell & Gresham, 1985; Clemen & Winkler, 1987; Clemen & Winkler, 1999) have shown that linear and logarithmic pooling perform as well as more complex models.

## Results

**Figure 1:**
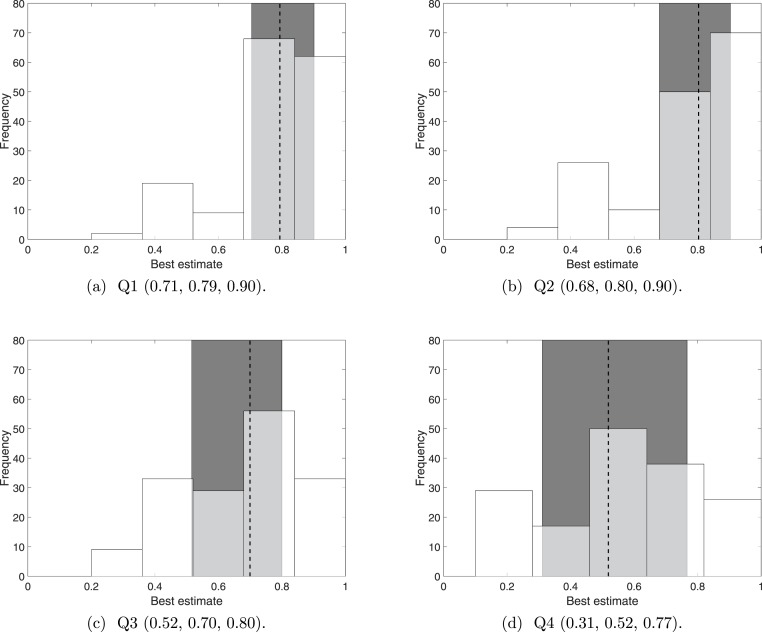
The distribution of ‘best’ estimates over 160 (5 experts scoring 32 sightings) responses, together with the 25th, 50th, and 75th percentiles. The dotted line indicates the 50th percentile (the median) and the shaded error indicates the interquartile range (the range between the 25th and 75th percentile). The 25th, 50th and 75th percentile values are provided under each plot.

We first considered the distribution of the raw data; that is, 160 (5 experts each judging 32 sightings) responses for each sighting (see Supplemental Information 3). When simply asked whether the sighting was correct (Q1), the responses follow a nearly identical distribution to responses on whether the sighting was distinguishable (Q2), see Figs. 1A and 1B. For both Q1 and Q2, to one decimal place, half the responses lie within the conservative range of 0.7 and 0.9, centred evenly around the median of approximately 0.8. Arguably distinguishability may not vary much, but the small interquartile range for Q1 raises questions about whether it is a true representation of the diverse sighting quality (see Supplemental Information 1). The broad nature of Q1 may make it more susceptible to behavioural aspects, such as question fatigue, than specific questions such as Q2, Q3 and Q4.

The additional two questions about observer competency (Q3) and verifiability (Q4) made the experts consider the sighting more sceptically. The experts generally considered the observers to be fairly competent, with Q3 having a median of 0.70, and no observers receiving a ‘best’ estimate of less than 0.2. The experts’ opinions of the observers competencies vary more than did their opinions on distinguishability (Q2), since the interquartile range (0.52 to 0.80) is approximately 130% that of Q2, see Fig. 1C.

Sightings of the Barbary lion are generally considered difficult to verify, with Q4 having a median of 0.52, with a range that almost spans the whole range of 0 to 1. In fact, the distribution resembles a normal distribution, see Fig. 1D.

**Figure 2:**
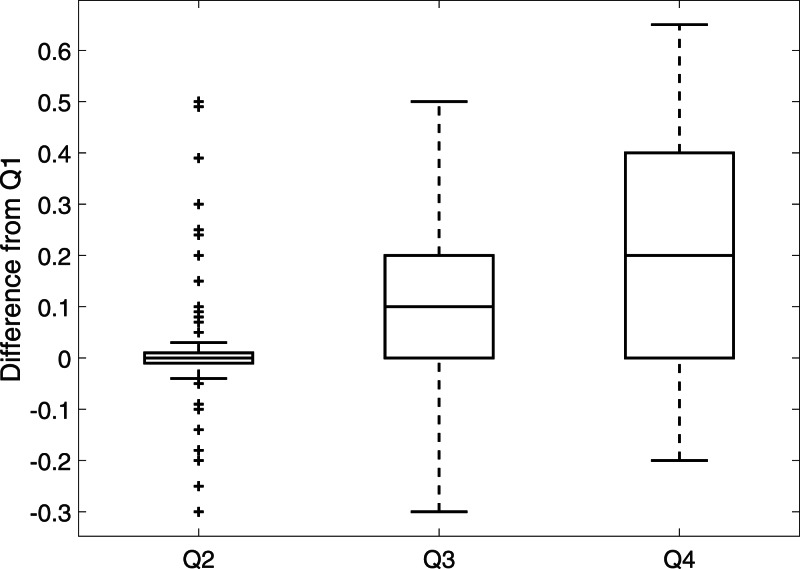
The dierence between best estimates for Q1 and Q2, Q3 and Q4 for 160 (5 experts scoring 32 sightings) responses.

The distributions of the ‘best’ estimates for all the questions show that asking experts Q1 only is insufficient: the range for Q1 is small, despite the experts acknowledging a huge range in verifiability (Q4). To further compare responses from Q1 to responses to Q2, Q3 and Q4, we take the difference between the best estimates for Q1 and the best estimates for Q2, Q3 and Q4, see Fig. 2. In agreement with Fig. 1, the median difference between Q1 and Q2 is zero with a minimum range around this average; whereas the median difference between Q1 and Q2 and between Q1 and Q3 indicates that Q1 receives a best estimate which is 0.1 higher than Q3 and 0.2 higher than Q4, with a considerable range in both these cases. It seems that left unguided, experts seem to only consider distinguishability (Q2) when deciding whether a sighting is valid.

### Pooling Q2–Q4

**Figure 3:**
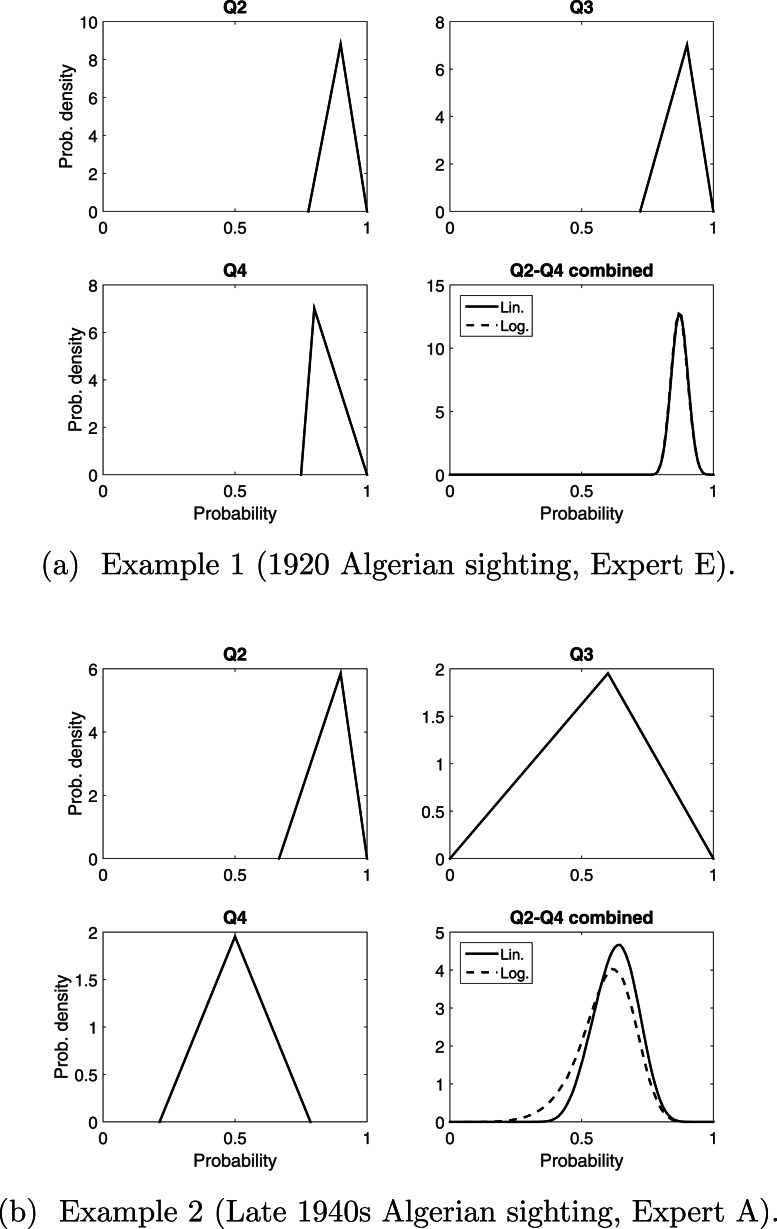
Two examples of pooling Q2–Q4 linearly and logarithmically. The triangle distributions are from responses to Q2, Q3 and Q4. In (B), “Q2–Q4 combined” is the consensus distribution from pooling these three triangle distributions. This process is carried out for all sightings for all experts.

Having established that asking Q2–Q4 more fully explores the different factors that might influence whether a sighting is valid, we need to consider how to combine these three responses. Linear and logarithmic pooling provide a very similar distribution to each other when the variation among Q2–Q4 are similar, see the example in Fig. 3A. When the variation among Q2–Q4 is larger, there is a more noticeable difference between the two pooling methods, especially in the bounds, see the example in Fig. 3B. These differences will be compounded once we pool the consensus distribution for each expert. For now we combine Q2–Q4 for each sighting, from each expert, and compare the resulting means (the peak of the distribution) from these 160 pooled opinions.

**Figure 4:**
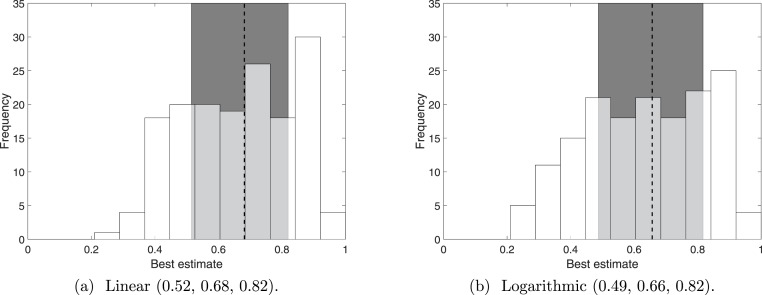
The distribution of the means from 160 distributions that combined Q2–Q4 (5 experts scoring 32 sightings). The 160 distributions resulted from pooling linearly or logarithmically. The dotted line indicates the median and the shaded error indicates the interquartile range.

We summarise the distributions from linear pooling and logarithmic pooling by their means. The distributions of these means (Fig. 4) are similar to each other, which is consistent with the examples discussed earlier (Fig. 3). More importantly, the pooled distributions are considerably different to the distribution of the ‘best’ estimate for Q1 (Fig. 1A). The median is reduced from 0.79 to 0.68 (linear pooling) or 0.66 (logarithmic pooling), and the interquartile range (in both linear and logarithmic pooling) is approximately 0.3, which is 150% of the interquartile range for Q1. The interquartile range, as with all the questions, is centred evenly around the median. The pooled interquartile ranges are smaller than the interquartile range for Q4 (0.46), demonstrating that neither pooling processes extend the variance of the resulting distribution (and thus loose certainty) in order to represent the pooled responses.

Therefore, because the Barbary Lion is a highly distinguishable species, simply asking whether a sighting is valid (Q1) can provide a high probability. Uncertainty in observer competency and sighting observation verifiability, which also account to sighting validity, may lower the probably, yet be overlooked unless explicitly included. Should a user prefer to keep distinguishability as a major factor, but still include observer competency and verifiability, the weighting would be changed (at present, these three factors are considered equal).

### Pooling experts

For each sighting the five expert opinions were pooled to provide a consensus distribution. We used three different pooling methods (averaging Q1, linearly Q2–Q4, and logarithmically Q2–Q4) using equal weighting and weighting based upon perceived expertise, giving a total of six different consensus distributions for each sighting. We split the sightings according to location: Algerian or Moroccan. Previous analysis (Black et al., 2013), which treats all sightings as certain, consider the locations separately and suggest that Barbary lions persisted in Algeria ten years after the estimated extinction date of the western (Morocco) population.

First we discuss the distributions for the individual sightings, where the expert opinions were pooled with a weighting function according expertise score. For our data, weighting by expertise score and weighting equally provided similar results to each other. Second, we compare the effect of weighting expertise, and the pooling methods, on the ‘best’ estimates only (the maximums from the distributions).

**Figure 5:**
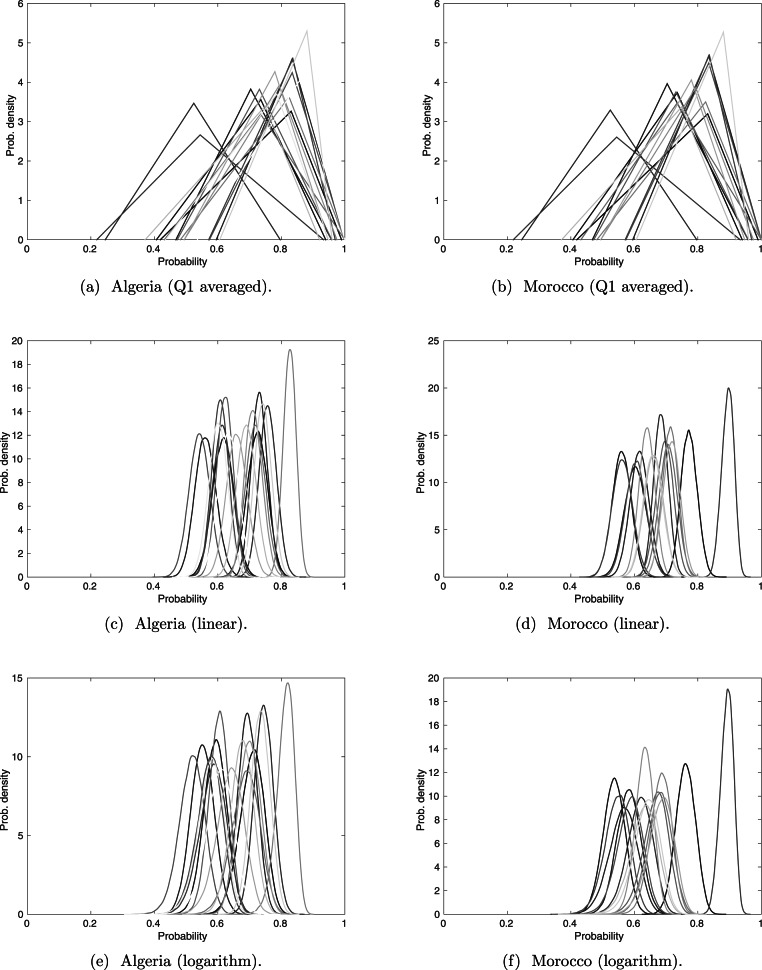
Sightings with experts’ opinions (weighted according to expertise) pooled linearly and logarithmically. The darker lines correspond to more recent sightings.

The averages of Q1, are represented as a triangle distribution, see Fig. 5. The range of these distributions covers a significantly larger range than do both linear and logarithm pooling. This may imply that Q1 received larger bounds than did Q2–Q4, but as previously seen (Fig. 3), linear and logarithm pooling tends to narrow the bounds, meaning that the pooled opinion is stronger than any experts’ opinion on its own. This follows the intuition that opinions from several experts provide a result that we have more confidence in.

There are slight differences between the linear and logarithm pooling. This is more noticeable in the Algerian sightings, where linear pooling gives stronger confidence in the sighting with the highest assessed validity probability. In cases like the Barbary lion, where no certain sighting has been formally recorded, the sighting with the highest assessed validity probability is treated as certain. Therefore, it is helpful that the most perceived valid sighting is reasonably distinguishable from the other sightings, as in the linear and logarithm pooling. We will discuss the ordering of all sightings under the different pooling techniques, but because of its particular significance for the Barbary lion, we first discuss the sighting with the highest assessed validity under each method.

According to the linear and logarithm pooling, the sighting with the highest validity in Algeria is in 1917. Yet the average from Q1 identifies 1911 is the most certain sighting. Similarly, in Morocco, the average from Q2 to Q4, irrespective of pooling method, identifies 1925 as the most certain sighting, whereas the average from Q1 identifies the 1895 sighting. This difference could have major consequences since extinction models usually require at least one ‘certain’ sighting.

With regards to the ordering of the rest of the sightings, we use a Wilcoxon rank sum test. The results indicate that linear and logarithm pooling rank the validity of sightings in a similar order (the *p*-value is 0.3 for Algerian sightings and 0.4 for Moroccan sightings), and neither of these rankings are similar to the ranking from Q1 (both comparisons to Q1 give a *p* value less than 0.01 for Algeria and Morocco).

**Figure 6:**
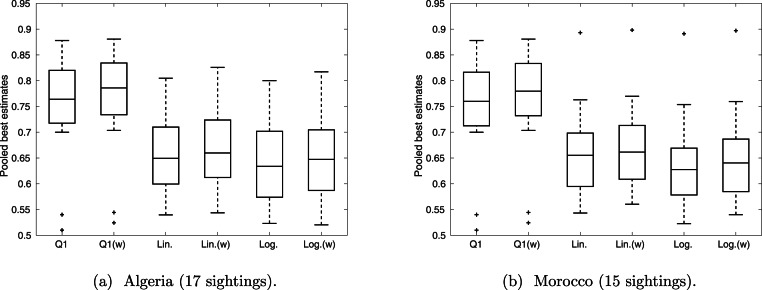
The distribution of ‘best’ estimates pooled over the expert opinions. The middle line marks the median over the sightings, the box represents the interquartile range, and the whiskers provide the range, excluding outliers (which are indicated by crosses).

Overall, linear and logarithm pooling provide similar outcomes (Fig. 6), with both providing a median valid probability of approximately 0.65 for all sightings. This is lower than the median valid probability under Q1, with an average pooling, which is over 0.75 for both Algeria and Morocco. Weighting experts according to perceived expertise shifts the median up in all cases, implying those that were perceived more qualified had stronger confidence in the sightings overall. This effect is more noticeable in Q1 than in Q2–Q4, implying that liner and logarithm pooling are more robust to variance in expertise.

## Discussion

In recent years there have been several extinction models that consider uncertainty of sightings in their calculations (Solow et al., 2012; Thompson et al., 2013; Jarić & Roberts, 2014; Lee et al., 2014; Lee, 2014). However, uncertain sightings are generally classed together (e.g., Solow et al., 2012), or grouped into smaller sub-groups based on degree of certainty (Lee et al., 2014). Generally these treatments gloss over the process of defining the probability that an uncertain sighting is valid. Therefore, there is a clear need to establish a formal framework to determine the reliability of sightings during assessments of extinction.

In the case of the Barbary lion, experts tended to provide estimates of the validity of a sighting in the region of 0.8 when asked the probability that the sighting in question was of a Barbary lion. The score is similar to those given when discussing distinguishability of the Barbary lion from other species in the region. This may suggest that when considering sightings of the Barbary lion the overriding factor is distinguishablity. To reduce the problem of one factor (such as distinguishability) overriding other potential issues in validating a reported sighting, a formal framework that considers observer competence and the verifiability of evidence is therefore required. Moreover, these three factors can be weighted if deemed appropriate.

Verifiability followed a normal distribution centred around 0.55. It would be interesting to apply this questioning technique to other species to establish whether sighting verifiability for other species can generally be modelled by a truncated normal distribution. If this shape repeatedly occurs, it is a question that experts could omit, and a normal distribution used instead. To perform such a test, and to establish the possible mean and variance, one would need a range of species with many sightings (such as the data set complied by Elphick, Roberts & Reed (2010)), and many experts who can provide their opinions.

Based on our assessment, it is reasonable to conclude that simply asking experts to provide a probability that a sighting is valid is not recommended. The pooled response from explicitly asking experts to score distinct elements that make up reliability of sighting results in a considerably more sceptical ‘best’ estimate (the mean of the distribution), with more variance in validity of sightings. The more sceptical ‘best’ estimates would result in a larger estimated probability that a species is extant from extinction models that account for uncertainty (Lee et al., 2014; Thompson et al., 2013; Lee, 2014), because an extended period of time without observing a certain observation is more acceptable.

The average of Q2–Q4 changed which sighting was considered most reliable when compared to the estimate from the omnibus question Q1. This is very significant in cases, like the Barbary lion series of reported sightings that we investigated, which did not have a well-accepted ‘certain’ sighting. Extinction models require at least one certain sighting, so in cases like the Barbary lion the most valid sighting would be treated as certain. This means that extinction could not have occurred prior to the date of that sighting. For example, using Q2–Q4 would prevent an estimate of extinction occurring before 1925 in Morocco, whereas Q1 would allow an estimate any time after 1895. In a Bayesian framework, one could place uncertainty around which estimate is the ‘certain’ one, which would alleviate this problem somewhat.

The decision as to whether to use linear or logarithm opinion pooling depends upon the situation. If the questioning process was followed as provided in this paper, linear pooling is recommended since it satisfies the marginalisation property, meaning that if we had pooled the experts before Q2–Q4 (instead of pooling Q2–Q4 first), we would arrive at the same distribution for each sighting, which seems intuitive. However, if experts or questions are continually being added at different times, then a logarithm pooling is preferred since it is externally Bayesian, meaning the consensus distribution can be updated incrementally. Alternatively, if only experts are added, but not questions, one could choose to pool Q2–Q4 using linear pooling, and pool the experts logarithmically. Or vica versa if the situation required. In these combination cases, the outcomes would lie somewhere within the small differences currently displayed by these two different pooling methods.

This framework may also reduce acrimony among observers who cannot provide verifiable supporting evidence. The suggested method uses group discussion, but ultimately experts provide their scores in private. The scores can be aggregated in an unbiased manner or weighted so that the opinion of the more experienced experts carries more influence.

Lastly, over time, the extinction probability output could enable decision-makers to forge a link between the process of sighting assessment and the process of concluding survival or extinction. The method is therefore less arbitrary than present methods such as decisions made on the basis of a vote by experts that is ascertained in a manner similar to Q1, or a final conclusion by the most senior expert. Furthermore, by identifying a probability, decision-makers are better able to apply the precautionary principle (Foster, Vecchia & Repacholi, 2000) on a data-informed basis rather than subjective assessment of available information.

## Supplemental Information

10.7717/peerj.1224/supp-1 Supplemental Information 1 Detailed information on the Barbary lion sightings

10.7717/peerj.1224/supp-2 Supplemental Information 2 The questions posed to the experts

10.7717/peerj.1224/supp-3 Supplemental Information 3 The experts’ responses
