# Supplementary material for: Assessing uncertainty in sighting records: an example of the Barbary lion
Source: PeerJ. 2015 Sep 1;3:e1224. doi: 10.7717/peerj.1224 (PMC4562256; doi:10.7717/peerj.1224)
Supplement: Supplemental Information 1 [file peerj-03-1224-s001.pdf]

Supplementary material 1. Last sightings of lions in North Africa from 1956 to 1895 (most recent first), including map of the distribution of sightings, provided to the panel of experts for assessment

Points raised during expert meeting are in Red

MOROCCO 1895-1942 (*Rif Mountains, Anti Atlas, Middle Atlas, High Atlas*) 15 observations since 1895. Sightings 21-7 (**M**).
ALGERIA1898-1960 (*Ksour Mountains, Saharan Atlas, Tell Atlas, Ouled-Nail, Aurès Mountains*). 17 observations since 1898 (Sightings 149-133 **A**)

Main lion areas in Algeria are described by leading writers (Gerard, 1859; Fenech, 1867; Margueritte, 1869) as woody mountainous landscapes around:

1- Mahouna valley and gorges- Saf Saf Valley –Annaba
2- Aurés Mounts (Batna-Khenchela)
3- Theniet el Had, Zaccar, Djebel Dira with connections to the Djurdjura mountains

**Obs**	**Year / Location**	**Sighting Type**	**Original source & Citation**	**Details – page 1 of 8 Note all map extracts for quick reference**<br>**Full map in Figure 1 on page 9**
149<br><br>A	1956<br>Beni Ourtilane (North of Setif)	Single lion observed	Local people on a bus [65]	Some persons from the Beni Ourtilane (Beni Uwarthelan) hamlet, about 60 miles north of town of Sétif (Seteef), informed me that around 1956 they were among a group of people travelling by bus when they saw a lion, a large male, at the edge of an oak forest, not far from the hamlet. They got so excited that the driver stopped the bus and, for a moment, everybody admired the majestic cat. They had been able to locate the lion because the driver was slowly riding, for the mountain path was in bad state and full of rocky twists and turns. Today, some of these persons, still living, are glad to tell for any occasion the encounter so well engraved in their memory.<br><br>The bus connection service from Sétif (Seteef) to Beni Ourtilane began in 1956 and stopped in 1958 due to the intensification of war.<br><br>The notion that this was a male lion (suggesting a mane) is important as verification of the species (only sexually dimorphic big cat). The sighting itself was not fleeting; in fact people had a period of time to look at and confirm what they saw. More than one person made the sighting. The area is very remote, so although there is a possibility that it was an escaped /released captive animal that is of low probability. The sighting was vivid and memorable for the people. It would be possible to re-interview some people.
148<br><br>A	Late 1940s North Setif	Group of lions observed	Interview local people<br><br>Difallah, pers. comm.	And in the vast hilly wilderness extending from North Sétif (Seteef), northwards to Amizour Stream, eastwards to the Biban mountain chain (This wilderness is located in the western part of northeast Algeria, roughly between Djurdjura (Jarjera) National Park and Babor Natural Reserve), a micro-population of lions seem to have been living on until the late 1940s. At least, one specimen from this group managed to survive up till the next decade…<br><br>This is a more general sighting, but confirmed with local people (who could be re-interviewed although some would now be old). The specifics of the sighting are less clear. The fact that ‘groups’ of animals were seen very strongly suggests lions (rather than leopards for example).
147<br><br>A<br>*	1943<br>Unknown location in Algeria	Single lion shot	Keeling, pers. comm. [66]	the last Algerian lion may have been shot in an unknown location as recently as 1943 (C. Keeling, pers. comm.).<br><br>A keen naturalist (Clinton Keeling) has spoken to people over many years in the region and heard about this 1943 shooting. There is little information other than the sincerity of Mr Keeling and his confidence in the people he spoke to which he then relayed to Dr N Yamaguchi.

Obs	Year / Location	Sighting Type	Original source & Citation	Details – page 2 of 8
21<br><br>M	1942<br>Tizi-n-Tichka pass (Marrakesh-Tadderte)	Solitary lion, shot	Minet, J.<br><br>[5] Cuzin	un lion a été abattu près de Taddert (versant nord du Tizi n'Tichka) en 1942 (J. Minet, com. pers.), ce qui constitue la dernière mention de l'espèce au Maroc<br>***Translation***<br>a lion was shot close to the town of Taddert (on the northern side of Tizi n' Tichka mountain pass) in 1942 (J. Minet, com. pers.), which is the last mention of the species in Morocco . [Highest point of pass is 2260m (7410ft)]<br><br>F Cuzin is a thorough scientist who would use good methods to interview people. The sighting is reliant on Mr Minet’s credibility. The animal was shot, so a body was seen. There is a suggestion that the animal was female (since it was not reported as male) but this is not confirmed.
20<br><br>M	1939<br>Hassi Aggou vicinity	Observations of 2 lions<br><br>Two animals close to Hassi Aggou	Hunter told Monteil 1951<br><br>[5] Cuzin	Selon un chasseur local, Monteil (1951) a signalé près de Hassi Aggou en 1939 deux animaux appelé "guerzam", rapportés au serval, dévorant un mouflon. Le nom de "guerzam" rapporté au berbère "wagerzam" est ambigu: selon la région, il désigne le lion ou la panthère, alors que, selon M. Bensalem et M. Ennah (com. pers.), ce nom désigne le lion en dialecte sahraoui des Tekna. Par ailleurs, l'observation de serval dévorant un mouflon (alors que cette espèce est incapable de chasser des proies aussi grosses, et n'a jamais été signalée) est très suspecte. Enfin, la localité où ont été observés ces animaux présente une couverture végétale très réduite, ce qui constitue *a priori* un milieu très peu favorable au serval. Cette observation se rapporte donc très probablement au lion, signalé dans le même secteur.<br>***Translation***<br>According to a local hunter, Monteil (1951) reports in 1939 near Hassi Aggou of two animals called " guerzam", attributed as the serval, devouring a sheep. The name of "guerzam" reported in Berber as "wagerzam" is ambiguous: according to the area, it indicates the lion or the panther, whereas, according to Mr. Bensalem and Mr. Ennah this name indicates, in the Saharawi dialect of Tekna, the lion. In addition, the observation of a serval devouring a sheep (since this species is unable to hunt down such large prey, and has never been reported , it being a carrion feeder) is very suspect. Lastly, the locality where these animals were observed presents a very reduced vegetation cover, which constitutes a very unfavourable habitat for the serval. This observation thus refers most probably to the lion, which was previously seen in this vicinity.<br><br>More than two animals are seen which infers a group living species (i.e. lion). Monteil’s original account merely reports the animals. Cuzin checked the local dialect used in this account, which suggest the word ‘lion’. Eating a large animal (sheep) strongly suggests lion rather than serval. Southern arid location does not discount lions from Senegal a location separated by perhaps 1000 miles /1600km (an area of 100,000 square miles) of the Western Sahara – a severely arid, desolate habitat, making the possibility less likely. Lions were seen here previously (below)
19<br><br>M	1935<br>Hassi Aggou and Hassi Tighissit between Assa –and TanTan	Lion group obs. at water points<br><br>Lions seen near water points	Bensalem, M.; Ennah, M.<br><br>[5] Cuzin	*en région saharienne, le lion existait jusqu'en 1935 à Hassi Aggou et Hassi Tighissit (région entre Assa et Tan Tan), près des points d'eau (M. Bensalem et M. Ennah).*<br><br>***Translation***<br>in the Saharan region, the lion existed until 1935 in Hassi Aggou and Hassi Tighissit (region between the towns of Assa and Tan Tan) near water points (Mr. Bensalem and Mr. Ennah, pers com.).<br>Cuzin (a credible and methodical scientist and interviewer) interviewed these two people who gave their own accounts of lion in this area. A group was observed. This location is at the edge of the maghreb ecosystem.

Obs	Year / Location	Sighting Type	Original source & Citation	Details – page 3 of 8
146<br><br>A	1935<br>Djebel es Somm (Djebel Amour)	Single lion obs.	Hamami Bachir<br><br>Interview by Fellous	Male lion attacked a camel<br>A boy herder (Mr Hamami then aged 5-6yrs) sees an animal…a big, big jackal with hair on his head … attacking his female camel. He runs to tell family - they tell him it is a lion. The male lion was then shot by the villagers after it attacked their female camel<br><br>A direct interview by an experienced, methodical interviewer. The information was volunteered by the witness. A compelling description from an observer who had no preconception. An animal capable of attacking a camle can only be a lion. The region is very remote (in the Saharan Atlas, so there is no chance that this could be a captive release animal. Local people pursued the animal and shot it, confirming the boy's sighting. Mr Bachir could be re-interviewed for verification if required.
145<br><br>A	Winter 1935<br>Boussam (Menaceur -Zaccar mounts)	Single male lion shot	B. Farid<br><br>Interview by Fellous	Male lion shot by the villagers after attacking a cow.<br>Account by Bounaceur Farid<br><br>A knowledgeable local person (Mr Farid) told the account of how a rich Frenchman offered money to the local villagers for retrieval of the skull and skin of the lion. The story of the Frenchman's money and the controversy surrounding how the money was used by the community is a story that still causes debate there today (more than the fact that a lion was shot for the money!). This adds credibility to the authenticity of the story. The interview was conducted by a thorough scientist using good interview methods.
144<br><br>A	1930s<br>DjebelDirah (Sour El Ghozlane)	Solitary lion shot	Mr Kalem Interview Fellous	Kalem, pers. comm.<br><br>This person (Mr Kalem) clearly recalled reading an account in a 1970s local newspaper of a lion being shot in the area in 1935 as it related to his own local area. A copy of the paper article has not yet been found, nor the original source of the story. The interview was conducted by a thorough scientist using good interview methods.
18<br><br>M	Summer 1930<br>Toubkal massif (now a NP)	Lion group Obs. at 3000 m<br><br>Lions seen by locals at 3000m	Local residents<br><br>[5] Cuzin	les habitants affirment qu'en 1930, des lions montaient en été jusque vers 3000m d'altitude dans le massif du Toubkal<br><br>***Translation***<br>[Local] people say that in 1930, lions went up in summer months up to around 3000m [9800 ft] in the massif of mount Toubkal<br><br>Cuzin (a credible and methodical scientist and interviewer) interviewed these people.
17<br><br>M	1930<br>Ouiouane area (Ain Leuh)	Lion group<br><br>Obs. a few lions, tracks	[8] Hemmer<br><br>[11] Panouse JB (1957)	Tracks seen in Ain Leuh (Ouiouane region) (Panouse)<br>"The last reference dates around 1930 in the Ouiouane area (literature compilation by Hemmer from Guggisberg; 1960 and Mazak; 1970). "<br>[58] Panouse JB (1957) Les mammifères du Maroc. Trav Inst Sci Chérif Sér Zool nu5. Rabat. 206 p.,<br><br>Panouse is a credible researcher and biologist. As several animals were seen around this time it suggests lions rather than leopards.

Obs	Year / Location	Sighting Type	Original source & Citation	Details – page 4 of 8
143<br><br>A	1930<br>North Setif	Lion Group Observed	Interview local people [65]	The group of animals implies lion rather than leopard. The region is known as habitat for lions in earlier decades/centuries. The interviewer is not a scientist but a writer and professor of linguistics. The interviews were conducted before 1994, so many of the people could now be dead and accounts would be more difficult to verify.
142<br><br>A	1930<br>Guenzet-Babor Mount (North Setif)	Solitary lion observed	Interview local people [65]	As above. The region is known as habitat for lions in earlier decades/centuries. The interviewer is not a scientist but a writer and professor of linguistics. The interviews were conducted before 1994, so many of the people could now be dead and accounts would be more difficult to verify.
141<br><br>A	Late 1920s Between Ain Talawane and Ain Roua (Setif)	Lion Group Observed (in spring)	Report by old man<br><br>Difallah, pers. comm.	Reported in 2002 by an old man.<br><br>Lions were seen when the old man himself used to take his animals along a route into the town for trading. The traders would often have to shout at the lions to discourage them from the trail, near to a spring where the animals took water. The interviewer is an Algerian with interests in the natural history of the region. The old man is now most likely dead (interviews were in the last 10+ years).
16<br><br>M	1925<br>Atlas Mountains (on the Casablanca -Dakar flight)	Solitary lion<br><br>Male lion seen from the air	Photograph Flandrin, M.<br><br>[10] Black (2008)	The precise location of the aerial photograph on the Casablanca-Dakar route (Fig. 2) is unknown, however our research has identified that it was taken in 1925 when flights commenced [38]. A postcard edition of the image has recently been discovered with the caption ‘‘Un lion photographie´en avion dans l’Atlas’’ (Fig. 3). Since this predates discussions on the extermination of lions in the region [19], its significance at that time was then unknown. The photograph by Flandrin is the last known image of a wild Barbary lion.<br><br>Figure 3. A lion seen in the Atlas Mountains, during a flight on the Casablanca-Dakar air route. The photograph taken by Marcelin Flandrin in 1925 is the last visual record of a wild ‘Barbary’ lion of North Africa. Black et al (2013)<br><br>Note: Fabrice Cuzin (pers comm with A. Fellous) is suggesting that the flight used in that time the coastal side of the Atlas mountain range (no radar in that time) Cuzin suggests also that it can be along Telfenay area , and in between Agadir, Essaouira...til Safi, where this kind of falls (calcarenite) can exist with sand in the surrounding...<br><br>A copy of the postcard hand dated and postmarked 1926 is in the possession of the authors. The flights in the region only started in 1925 and Flandrin was the official photographer hired to record the new routes by the airline. A scan of the postcard has been seen by historians and is thought to be an authentic image, even if slightly enhanced for publication purposes. The photographer did not make any similar photographs of lions or other animals form the air. It was not known at the time that the animal might be extinct or near extinction (no publications before 1932 suggest extinction, so although unusual the photograph would not have been considered sensational. The animal is behaving in a reasonable way considering it was being overflown by a slow, distant aircraft. There is a small chance it could be a lion from Senegal (1000km away). The region is remote.

Obs	Year / Location	Sighting Type	Original source & Citation	Details – page 5 of 8
15<br><br>M	1922 Middle Atlas mountains	Solitary lion<br><br>Shot	[56] Yadav PR	[56] Yadav PR (2004) Vanishing and endangered species. New Delhi: Discovery Publishing House<br><br>This is a generic ‘encyclopaedic’ reference, is not very specific. The publisher is not a credible source and the material appears copied from Day (1981). The 1922 date does not appear verified in any other sources, although it is widely presented as be ‘true’ in blogs, websites and newspaper articles.
14<br><br>M	1920 Azrou vicinity	Solitary lion<br><br><br><br>Obs. of a single male	[8] Hemmer H (1978)<br><br>[55] Grzimek B (1975)	*“in 1920 a single male lion was seen in the vicinity of Azrou”* (Hemmer 1978)<br><br>[20] Hemmer H (1978) Grundlagen und derzeitiger Stand des Zuchtprogrammes zur Ru¨ckerhaltung des Berberlo¨wen (Panthera leo leo). In: Seifurt S, Mu¨ ller P, editors. Congress Report, 1st International Symposium on the Management and Breeding of the Tiger, 11th and 12th October 1978 in Leipzig, Abb. 1. Zoological Garden. Leipzig: International Tiger Studbook. 65–72.<br>[55] Grzimek B (1975) The lion, In Grzimek, B. (Ed.), Grzimek’s Animal Life Encyclopedia, vol. 12, Mammals III.Van Nostrand Reinhold, New York, 353.<br><br>Although this is again a somewhat generic reference based on bibliography, it is not very specific. However it is clear that it is a male (and therefore must be a lion rather than another species). The 1975 reference is a generic encyclopaedic comment, rather than a clearly sourced account.
140<br><br>A	c.1920 Djebel Tameda,	Solitary lion – last in Saharan Atlas	Gueniche Ahmed	One shot at Djebel Tameda, south of Boussemghoun (Algeria), the last in the Saharan Atlas; between High and Tell Atlas.<br><br>Account by Bahmane L to Fellous<br><br>Mr Bahmane is an experienced former ranger in the region, who spoke to a scientist who used good interview methods. The animal was memorable for being the last shot in the remote region.
13<br><br>M	Winter 1917 Oued Ifrane	Solitary male lion obs.	[6] Lavauden	Lavauden L (1932) Les grands félins de l’Afrique du Nord et leur disparition. Le Chéne. Soc. Forestière Mediterranéenne et Coloniale. N°4 Janvier: 208–234.<br><br>This author is a credible scientist who has published many reviews of north African fauna. The account mentions a male (suggesting mane) and therefore a lion.
139<br><br>A	1917 Biskra (lion probably from Aurès ranges)	Solitary Old lion obs.	[64]	*“In the early 20th Century an old spotted male lion with a magnificent mane ventured into Biskra oasis, apparently coming from the Aurès, to settle there. The natives of the city daily carried to the kubba food for the lion, and every one called the animal ‘a Marabout.’ It turned out to be blind and, when it died ten years ago, a magnificent funeral procession accompanied it to the place of its eternal rest.”*<br><br>Spotted lions (spots on legs in particular) are seen photographs from in North Africa, so it does not presuppose a leopard. Also a mane is mentioned. Aures is only a few tens of kilometers from Biskra. The author professes to having seen the lion himself when visiting the oasis. It could be a tamed Marabout lion; photographs of a tame lion in Biskra are known from the early 1900s (it may have originated from the wild).

Obs	Year / Location	Sighting Type	Original source	Details – page 6 of 8
138<br><br>A	1912<br>Ain Sefra	Solitary lion Shot	Khazene, A	Ain Sefra, Saharan atlas (Algeria) Account by Khazene to Fellous,pers. comm.<br><br>Mr Khazene runs a blog for an NGO and got the information from another person. No other details.
137<br><br>A	1912<br>Bejaia area	Solitary lion Shot	[63] De Smet	Apart from the location, and the shooting of a single animal there is little detail, although the source (De Smet) is a credible scientist.
135<br><br>A	1910-12<br>Zaccar-AinTorki	Solitary lion obs.	Mrs Dedreuil-Paulet<br><br>Difallah, pers. comm.	In the years 1910-12, in northwest Algeria, between Ain Torqui village (formerly Marguerite) and the Tizi-Ouchir (Teezy Usheer) col, that is to say between Miliana and Hammam-Righa, two ladies – Mrs Dedreuil-Paulet and a friend of her – were going out for a walk when they encountered a lioness; the animal has been conspicuous in this region of the Zaccar-Gharbi (West Zaccar) for a few days devouring some head of cattle and a calf, and young shepherds also had seen it.<br><br>The interviews were by a local Algerian naturalist. The account suggests more than one observer and the animal had been seen several times. A lioness is mentioned, which suggest firmly that the animal was considered a lion, albeit without a mane (it could potentially have been a large juvenile male, although less likely to have been able to kill a cow perhaps). The people could perhaps be re-interviewed although now most likely dead.
12<br><br>M	1911<br>Zaián forests, Beni Mgild (Khenifra)	Lion group<br><br><br>Lions	Sultan Moulay Hafid to [1] Cabrera<br><br>[3] Guggisberg (1963) Simba: the life of the lion.	**Continued description from Cabrera**<br>Segun me comunico verbalmente, hace algun tiempo, el ex sultan Muley Hafid, en los ultimos anos de su reinado, es decir, justamente en la epoca en que escribia Engell, solo quedaban en Marruecos algunos leones que vivian en los bosques de los Zaian y de los Beni Mguild.<br><br>***Translation from Spanish - Cabrera (above)***<br>From my own verbal communication [I was informed], some time ago, the former sultan Moulay Hafid, in the last years of his reign, ie just at the time Engell's account was published, in Morocco there were only a few lions who lived in the forests of Zaian the territory of the Beni Mguild (below).<br>Guggisberg - *"In 1911 only a few were still found in Morocco…in the forests of Zaian and Beni Mgild. According to some rumours some may have survived up to 1922 in the Middle Atlas, while in the High Atlas they possibly held out even longer."*<br><br>Should we accept the comments of a Sultan (exaggeration?), would he have visited the area (when younger to hunt?). The area is specific and the tribe mentioned is from that region.

Obs	Year / Location	Sighting Type	Original source & Citation	Details – page 7 of 8
11<br><br>M	1911<br>Middle Atlas mountains	Lion group<br><br>Obs.	Local hunter to Engel<br><br>[1] Cabrera	En 1911, Engell incluyo toda la region del Atlas Medio entre los paises en que el leon era todavia frecuente, considerandolo como raro en toda la zona inmediata alrededor de dichas montanas; pero los datos de este autor no deben admitirse sin ciertas reservas desde el momento que tambien afirma la presencia de la fiera, en la misma fecha y como "ziemlich häufig", en la parte oriental de Argelia y en Tunez, siendo asi que en ambos paises se mato el ultimo leon en 1891, o sea veinte anos antes.<br><br>***Translation from Spanish***<br>In 1911, Engell included the entire Middle Atlas region between the countries as [an area] in which the lion was still prevalent, considering it rare throughout the immediate area around these mountains, but this author's data [*i.e. Engell's view*] should not be admitted without some reservations from the moment it also affirms the presence of the beast, on the same date as "quite often" in the eastern part of Algeria and Tunisia, being so in both countries were killed the last lion in 1891, or twenty years before[*].<br>[* Black et al list 12 sightings in eastern Algeria after the last account of a lion killed in Tunisia and Algeria up to Engell's comments in 1911]<br>Cabrera's rejection/reservation of Engell's accounts is misplaced. Engell is correct in stating that lions persisted in Algeria in 1911, so his sources in Morocco may also be better that Cabrera assumes.
136<br><br>A	1911<br>Aurès Mounts	Lion group<br>Shot, Male, female	Sassorossi family<br>[61?]	**Source (?) :**<br>Martin JP (2006) Le Belezma: au forgeron de Batna. Paris: L'Harmattan<br>An account in a recent book. Killing of pairs (male and female) is described in the 1890s newspaper accounts – from thes animals the cubs were often taken for private sale. It suggest a clear identification, but difficult to test, although a lion skin might be kept in a French manor house or chateau…
134<br><br>A	1900's<br>Beni Salah (Chréa NP vicinity)	Lion group<br>obs.	Residents of Beni Salah<br><br>[62]	Desparmet (1939): Some inhabitants of Beni Salah (Blida vicinity) have seen (lions) still living in the beginning of the XXth century.<br><br>Not very specific except for the location, an area with the correct and advantageous habitats for lions. A group of animals suggests lions and not leopards.
10<br><br>M	1901<br>Budaa woods (Azrou)	Lion group<br><br>Obs., frequent lions	Marquis of Segonzac<br>[1] Cabrera A (1932) Los mamıferos de Marruecos. Seria Zoologica Madrid: Trabajos del Museo Nacional de Ciencias Naturales	*Sin embargo, según el marqués de Segonzac, todavía en 1901 eran los leones y las panteras "hôtes passagers mais fréquens" del bosque de Budaa, cerca de Azrú. Dicho explorador conoció en esta población al más famoso cazador de leones de la zona, y refiere que estas fieras eran en el invierno el terror de las gacelas y otros rumiantes que descendían a los valles huyendo de las nieves.*<br>***Translated from Spanish (Cabrera 1932)***<br>However, according to the Marquis de Segonzac, even in 1901 lions and leopards were "hôtes passagers mais fréquens" ('frequent guest visitors') to the forest of Buda, near Azru. This explorer met amongst the locals the most famous lion hunter in the area, and states that these beasts were in the winter the terror of gazelles and other ruminants that descended to the valleys to escape the snow.<br><br>***Other quote from Segonzac 'Travels in Morocco'*** (Zimmerman, 1905): *"Nous n'avons relevé aucune trace de lion ni de panthère, hôtes passagers mais fréquents de ces parages. On n'a tué qu'un gros serpent si bien haché de coups de fusil que je n'ai pu l'identifier."*<br>***Translation:*** We have not seen or boars, or gazelles, or monkeys, common game of the forest. We found no trace of lion or panther, but they are frequent visitors to the vicinity. One had killed a big snake which was so gashed and gored that I could not identify [the species].<br>This is a passing reference which appears in a couple of accounts, from the travel accounts of people on the Marquis' trip. The mention of prey species suggests that the local hunter had a good level of knowledge. He also mentions both leopard and lion, suggesting both species, rather than one or another in error.

Obs	Year / Location	Sighting Type	Original source & Citation	Details – page 8 of 8
9<br><br>M	c.1900 Djebel Ebrit (Ain Leuh-Timahdit)	Lion group obs.	[6] Lavauden L (1932)	Lavauden L (1932) Les grands félins de l'Afrique du Nord et leur disparition. Le Chéne. Soc. Forestière Mediterranéenne et Coloniale. N°4 Janvier: 208–234.<br><br>The scientist is a credible source of information on North African mammals. Group of animals strongly suggests lion.
8<br><br>M	c.1900 M'Hamid, south of Zagora (Morocco-Algeria border)	Lion group obs. close to water points	Local inhabitants<br><br>[5] Cuzin F (2003)	[en région saharienne, le lion existait jusqu'en 1935 à Hassi Aggou et Hassi Tighissit (région entre Assa et Tan Tan), près des points d'eau (M. Bensalem et M. Ennah, com. pers). **Au début du siècle, des habitants de M'hamid ont signalé l'existence de lions près des points d'eau permanents sur le revers nord de la Hamada, sur la frontière algéromarocaine]**<br><br>***Translation***<br>[in the Saharan region, the lion persisted until 1935 in the area around Hassi Aggou and Hassi Tighissit (the region between the towns of Assa and Tan Tan) where they kept near near water points (pers Mr. Bensalem and Mr. Ennah, com.). **At the beginning of the century, the inhabitants of the town of M'hamid reported the existence of lions near permanent water courses on the north side of the 'Hamada' [**high, largely barren, hard, rocky desert plateaus with little sand] **on the Algeria-Morocco border].**<br><br>Cuzin F (2003) Les grands mammiferes du maroc-meridional (Haut Atlas, Anti Atlas et Sahara): distribution, écologie et conservation. PhD thesis, Universite Montpellier.<br><br>Interviews by Cuzin are thorough and follow good methods about 10 years ago. The people could be re-interviewed. The area is very remote. Sightings near water points would be expected.
133<br><br>A	1898 Djebel Amour	Lion group	[60]	Numerous lions<br>Anon. (1898) La revue hebdomadaire. Paris: Librairie Plon. 378 p.<br><br>The source was written in 1898, so it is not clear if the sightings relate to contemporary accounts form that year, or from earlier years in the decade. The source could be located and verified if required.
7<br><br>M	1895 Rif mountains	Solitary lion<br><br>Last one killed in the Rif	[26] de Planhol, 2004	The last lion in the Rif mountains was killed in 1895.<br><br>Reference de Planhol X (2004) *Le paysage animal. L'homme et la grande faune: une zoogéographie historique*. Fayard, Paris, France.<br><br>The encyclopaedic reference can nevertheless be re-examined (original sources). A shot animal suggests a lion was identified from its body.

Observations of lions in North Africa 1895 - 1960 Grey shading indicates Mediterranean forest, woodland and scrubland ecosystems; circular markers denote locations of major human population centres. Dashed lines indicate national boundaries. Triangular markers indicate lion sightings, with sightings 7–21 in Morocco (all at western longitudes) and sightings 133 – 149 in Algeria (eastern longitudes). Sighting 147 is not depicted (location in Algeria unknown). Sighting 16 was recorded somewhere on the air route indicated by the dotted line in Western Morocco.

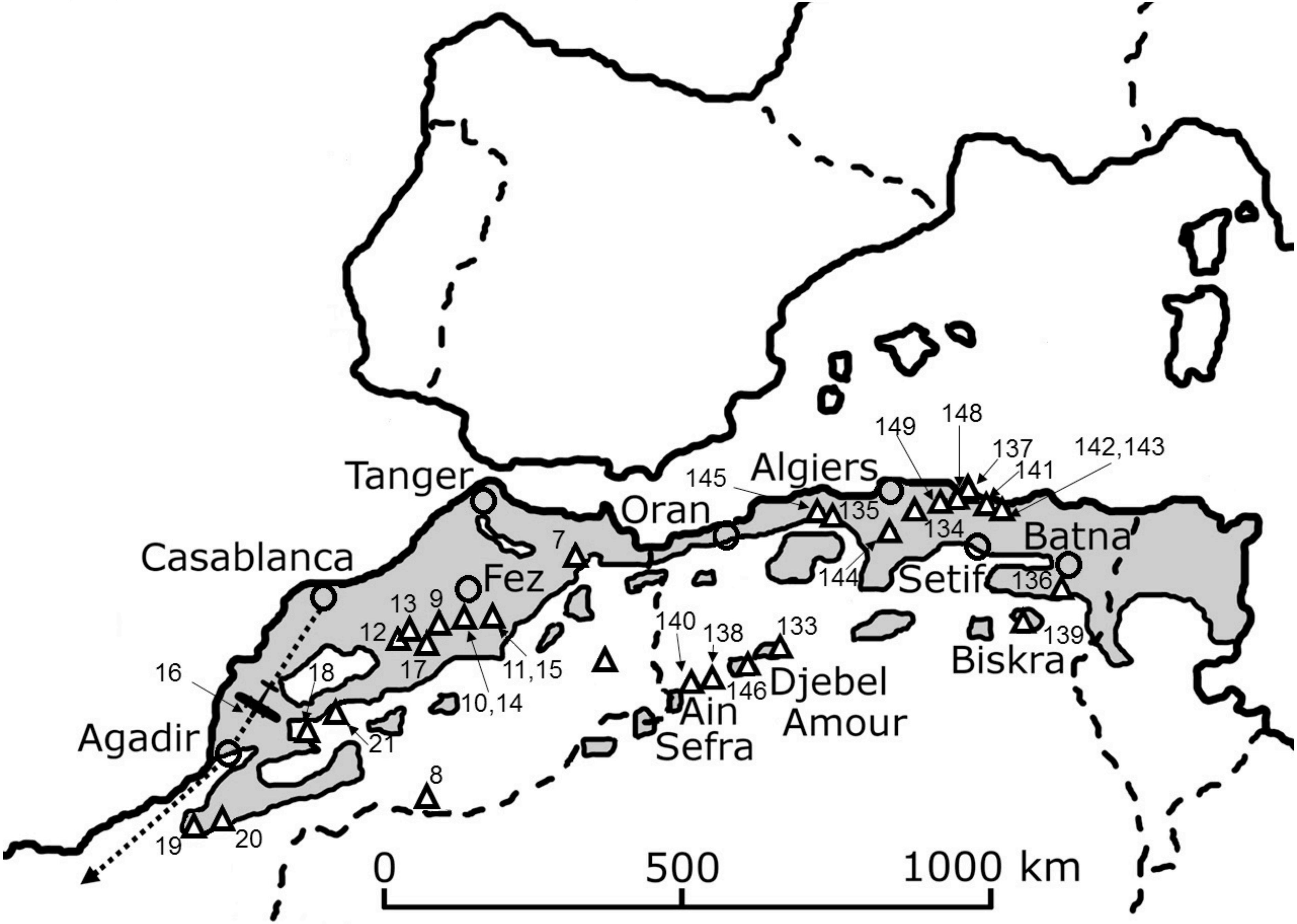

References

[1] Cabrera, A. (1932) Los mamíferos de Marruecos. Seria Zoologica. Madrid: Trabajos del Museo Nacional de Ciencias Naturales.

[2] Drummond-Hay, J.H. (1861) Morocco and the Moors. Western: its tribes and savage animals. London: Murray.

[3] Guggisberg, C.A.W. (1963) Simba: the life of the lion. London: Bailey Bros and Swinfen.

[4] Schnitzler, A.E. (2011) Past and present distribution of the North African-Asian lion subgroup: a review. *Mammal Rev* 41: 220-243.

[5] Cuzin, F. (2003) Les grands mammiferes du marocmeridional (Haut Atlas, Anti Atlas et Sahara): distribution, écologie et conservation. PhD thesis, Université Montpellier.

[6] Lavauden, L. (1932) Les grands félins de l'Afrique du Nord et leur disparition. Le Chéne. Soc. Forestière Mediterranéenne et Coloniale. N°4 Janvier: 208-234.

[7] Grzimek, B. (1975) The lion, In Grzimek, B. (Ed.), Grzimek's Animal Life Encyclopedia, vol. 12, Mammals III.Van Nostrand Reinhold, New York, pp. 353.

[8] Hemmer, H. (1978) Grundlagen und derzeitiger Stand des Zuchtprogrammes zur Rückerhaltung des Berberlöwen (*Panthera leo leo*). In: Seifurt S, Müller P, editors. Congress Report, 1st International Symposium on the Management and Breeding of the Tiger, 11th and 12th October 1978 in Leipzig, Abb. 1. Zoological Garden. Leipzig: International Tiger Studbook. pp. 65-72.

[9] Yadav, P.R. (2004) Vanishing and endangered species. New Delhi: Discovery Publishing House.

[10] Black, S. (2008) Investigating the feasibility of reintroducing lions (*Panthera leo*) as a flagship for the Moroccan Atlas Mountains. MSc thesis, University of Kent.

[11] Panouse, J.B. (1957) Les mammifères du Maroc. *Trav Inst Sci Chérif Sér Zool* n°5. Rabat. 206 p.

[12] Playfair, R.L. (1877) Travels in the footsteps of Bruce in Algeria and Tunis. London: G. Kegan Parl & Co.

[13] Daumas, E. (1855) Moeurs et coutumes de l'Algérie. Paris: Sind bad Bibliothequearabe.

[14] Roches, L. (1904) Dix ans à travers l'Islam, 1834-1844. Paris: Librairie Academique Didier.

[15] Carette, E. (1844) Exploration scientifique de l'Algérie pendant les années 1840-1841-1842. Paris: Imp Roy Tome II: 355.

[16] Gerard, J. (1864) Chasse au lion. Cie Editeurs. Québec: JN Duquest.

[17] Scott, K.C. (1842) A journal of residence in the Esmailla of Abd-El-Kader. London: Whittaker & Co.

[18] Fenech, E.V. (1867) Récits et chasses d'Algérie. Typographie Denis Aimé.

[19] Margueritte, A. (1869) Chasses de l'Algérie et notes sur les arabes du Sud. 1ère edition. Paris: Jouvet Editeurs.

[20] Bloch, A. (2002) Miliana par les textes. Algiers, Editions Zyriab.

[21] Daumas, E. (1845) Le sahara Algérien. Paris: Langlois & Leclercq.

[22] Anon. (1845) Journal des haras, des chasses et des courses de chevaux. Imprimerie Parent Tome III. Bruxelles.

[23] Kennedy, J.C. (1846) Algeria and Tunis in 1845. Vols I & II. London: H Colburn Publisher.

[24] Gerard, J. (1892) Le tueur de lions. Paris: Paris librairie Hachete & Cie.

[25] Saint Marie, C. (1846) Algeria in 1845. A visit to a French possessions in Africa. London: R Bentley.

[26] de Planhol, X. (2004) Le paysage animal. L'homme et la grande faune: une zoogéographie historique. Paris: Fayard.

[27] Palisser, G. (1982) Les lions et panthères dans le Sahel. *L'Algérianiste* n°20, Décembre, Narbonne, France.

[28] Lestiboudois, T. (1853) Voyage en Algérie, ou études sur la colonisation de l'Afrique française. Lille: Imp Danel.

[29] Saint Arnaud, A.J.L. (1864) Lettres du maréchal de saint Arnaud.1832-1854. 3 ème édition. Paris: M Levy frères.

[30] Gerard, J. (1859) La chasse au Lion. Paris: Paris Librairie nouvelle.

[31] Dufour, L. (1856) Notice sur un lion tué en Algérie. Examen necroscopique. Paris: H & C Noblet Imprimerie.

[32] D'Esschavannes, M.J. (1852) Revue de l'Orient, de l'Algérie et des colonies. Paris: Rouvé J Tome XII: 221-222.

[33] Bombonnel, C.L. (1893) Bombonnel, le tueur de panthères, ses chasses racontées par lui m�me. 7eme édition, Paris: Librairie Hachette.

[34] Anon. (1860a) The hunting grounds of the Old World. London: Sanders Otley & Co.

[35] Gastineau, B. (1863) Chasses au Lion et à la Panthère en Afrique. Paris: Paris Librairie Hachette.

[36] Blakesley, J.W. (1859) Four months in Algeria: with a visit to Carthage. London: MacMillan & Co.

[37] Dunant, J.H. (1858) Notice sur la régence de Tunis. Genève: Imprimerie Jules Fick.

[38] Anon. (1860) *Journal des chasseurs*. Vol. 24, 2eme sem, mai-octobre: 239-248.

[39] Thierry-Mieg, C. (1861) Six semaines en Afrique, souvenirs de voyage. Paris: M Levy frères.

[40] Bernard, A., Lacroix, N. (1906) L'évolution du nomadisme en Algérie. Alger: Edition Jourdan.

[41] Windham, W.G. (1862) Notes in North Africa: guide to the sportsman and tourist in Algeria and Tunisia. London: Ward & Lock.

[42] Guerin, V. (1862) Voyage archéologique dans la régence de Tunis. Paris: TI Plon.

[43] Baroli, M. (1967) La vie quotidienne des français d'Algérie (1830-1914) Paris: Librairie Hachette.

[44] Ormsby, J. (1864) Autumn rambles in North Africa. London: Longman & Green.

[45] Anon. (1936) Revue Chasse et pêche Nord Africaines. Mars n° 48: 7.

[46] Carteron, C. (1866) Voyage en Algérie. Paris: J Hetzel Librairie.

[47] Berard, V. (1867) Indicateur general de l'Algérie. Alger: Typo Bastide, 3eme edit. 600 p.

[48] Loche, V. (1867) Histoire naturelle des mammifères. Paris: Arthis Bertrand Editeur.

[49] Tounsi, G. (1998) Un lion surgit dans la tribu des Sinfita à Tenès. Evocation. *Journal Le Jeune Indépendant* N°14 du 25 au 31 mars. 24 p.

[50] Simmonds, P.L. (1877) Animal Products: their preparation, commercial uses, and value. London: Chapman & Hall. 416 p.

[51] Bourde, P. (1880) A travers l'Algérie. Souvenirs de l'excursion parlementaire, septembre-octobre 1879. Paris: G Charpentier.

[52] Gharaibeh, B.M. (1997) Systematics, distribution, and zoogeography of mammals in Tunisia. PhD thesis, Texas Tech University, Lubbock.

[53] Emmanuel, J. (2003) Maupassant, guy, sur les chemins d'Algérie (1881-1890). Paris: Magella & Cie.

[54] Lataste, F. (1885) Étude de la faune des vertébrés de Barbarie (Algérie, Tunisie et Maroc). *Actes de la Société Linnéenne de Bordeaux* 39: 129-289.

[55] Robert, G. (1891) Voyage à travers l'Algérie: notes et croquis. Paris: Imprimerie de G Rougier.

[56] Seurat, L.G. (1924) Zoologie forestière de l'Algérie. Alger: Direction des Forêts.

[57] Lavauden, L. (1926b) Les vertébrés du Sahara. Tunis: Imp A Guénard.

[58] Anon. (1893) Rubrique annonce. Le Chenil, n°12 du 23 mars: 137-138.

[59] Hanoteau, A., Letourneux, A. (1893) La kabylie et les coutumes kabyles. Tome I. Paris: Imprimerie Nationale.

[60] Anon. (1898) La revue hebdomadaire. Paris: Librairie Plon. 378 p.

[61] Martin, J.P. (2006) Le Belezma: au forgeron de Batna. Paris: L'Harmattan.

[62] Desparmet, J. (1939) Coutumes, institutions, croyances des indigènes de l'Algérie. Tome I, Imprimerie.Alger, La typo-litho and J Carbonel.

[63] De Smet, K. (1982) La disparition des grands fauves en Algérie. *Bull Forest Conserv Nat Alger* 1: 23-25.

[64] Ossendowski, F.A. (1927) The breath of the desert: the account of a journey through Algeria and Tunisia (translated by Palen LS). London: George Allen and Unwin.

[65] Haddadou, M.A. (1994) L'Algérie mystérieuse. Alger.

[66] Yamaguchi, N., Haddane, B. (2002) The North African Barbary lion and the Atlas lion project. *International Zoo News* 49: 321.
