# Supplementary material for: Assessing uncertainty in sighting records: an example of the Barbary lion
Source: PeerJ. 2015 Sep 1;3:e1224. doi: 10.7717/peerj.1224 (PMC4562256; doi:10.7717/peerj.1224)
Supplement: Supplemental Information 2 [file peerj-03-1224-s002.pdf]

Supplementary material 2

Instructions sent to the experts before the meeting

Before we meet I would like you to go through all the sightings and assign values between 0 and 1 using the method in the section below (Assigning values). For each sighting you will have 16 values. Please email these to me asap so that I can put them together and send them out to the group to show the distribution of values (d.l.roberts@kent.ac.uk). Note that this is anonymous in that I will not reveal your values to the group at the meeting.

At the meeting I would like you to do is, following the discussion of each sighting reconsider your previous assessments. We will discuss the evidence for each sighting and the values assigned prior to the meeting. I shall present the distribution and range of values for discussion. At the meeting I would like you to discuss the distribution of values and talk to why it may be so high or so low. Once we have finished the discussion you will have a chance to change your estimate following the protocol below (Assigning values).

Assigning values

Following the method of Burgman et al. (2011) and McBride et al. (2012), for each of the following I would like you to write down the following for each of the sightings based on a scale of 0 to 1, your

(a) upper (highest) estimate

(b) lower (lowest) estimate

(c) best (most likely) estimate

and finally

(d) how confident are you that the interval you provided contains the truth (provide an answer in the range of 50–100%)?

The four attributes I would like you to give estimates (upper, lower, best & confidence) for are the probability that

1. the sighting is a Barbary Lion - this I hope is relatively self explanatory.
2. the individual sighting is distinguished from other taxa - other species occur within the area the sighting was made, therefore how distinguishable is this species? Note that this is NOT based on the type of evidence you are presented with, i.e. a photo or a verbal account.
3. the observer is competent - how competent is the person who made the sighting at identifying the species. Note that if you are looking at a museum specimen or say a photograph then it is your own competence as you are the one making the observation i.e. saying that it is this species.
4. the sighting could be verified by a third party - this is essentially the quality of the sighting evidence. For example, a poor quality photo has the same ‘verifiability’ as a good photo, but a good quality photo, which is not blurred, would score higher. Further it may also score higher than say a second hand oral record, which could be verified from the point of view that someone else could ask the observer. Note that these are merely examples; the estimate you assign is up to you.
